# Signaling of the Complement Cleavage Product Anaphylatoxin C5a Through C5aR (CD88) Contributes to Pharmacological Hematopoietic Stem Cell Mobilization

**DOI:** 10.1007/s12015-017-9769-6

**Published:** 2017-09-16

**Authors:** Kamila Bujko, Sylwia Rzeszotek, Kai Hoehlig, Jun Yan, Axel Vater, Mariusz Z. Ratajczak

**Affiliations:** 1 Stem Cell Institute at James Graham Brown Cancer Center, University of Louisville, 500 S. Floyd Street, Rm. 107, Louisville, KY 40202 USA; 2 Aptarion Biotech AG, Berlin, Germany; 3 Department of Regenerative Medicine, Warsaw Medical University, Warsaw, Poland

**Keywords:** Complement cascade, Hematopoietic stem cells, Stem cell mobilization, C5aR-KO mice, Anti-C5a L-aptamer, AON-D21, PNH

## Abstract

Several mechanisms have been postulated for orchestrating the mobilization of hematopoietic stem/progenitor cells (HSPCs), and we previously proposed that activation of the complement cascade plays a crucial role in the initiation and execution of the egress of HSPCs from bone marrow (BM) into peripheral blood (PB). In support of this notion, we demonstrated that mice deficient in the mannan-binding lectin (MBL) pathway, which activates the proximal part of the complement cascade, as well as mice deficient in the fifth component of the complement cascade (C5), which is part of the distal part of the complement cascade, are poor mobilizers. To further narrow down on the exact mechanisms and the molecules involved, we performed studies in mice that do not express the receptor C5aR, which binds the C5 cleavage fragments, C5a and C5a_desArg_. We also employed the plasma stable nucleic acid aptamer AON-D21 that binds and neutralizes C5a and C5a_desArg_. We present evidence that mice deficient in C5aR or treated with AON-D21 are poor HSPC mobilizers, thereby establishing a critical role for the C5a/C5a_desArg_–C5aR axis in the mobilization process. While enhancing mobilization is of clinical importance for poor mobilizers, inhibition of the complement cascade could be of therapeutic importance in patients suffering from paroxysmal nocturnal hemoglobinuria (PNH) or acquired hemolytic syndrome (aHUS).

## Introduction

Hematopoietic stem/progenitor cells (HSPCs) reside in bone marrow (BM) niches. It is widely accepted that the alpha chemokine stromal-derived factor 1 (SDF-1)–CXCR4 axis and the vascular adhesion molecule 1 (VCAM-1)–very late antigen-4 (VLA4) integrin axis both play important roles in their BM retention [1]. On the other hand, it is well known that low numbers of HSPCs circulate in peripheral blood (PB) under steady-state conditions in a circadian rhythm-dependent manner, with the peak occurring early in the morning and the nadir at night [2]. In addition, the number of circulating HSPCs increases in PB in response to inflammation, strenuous exercise, and tissue/organ injuries [3–5].

The number of HSPCs circulating in PB may be increased up to 100-fold after administration of agents such as the cytokine granulocyte colony stimulating factor (G-CSF) and the small molecular CXCR4 antagonist AMD3100 (Plerixafor). This process is known as “pharmacological mobilization” [6] and can be used to obtain HSPCs for hematopoietic reconstitution in clinical settings.

On the other hand, several mechanisms for orchestrating the mobilization of HSPCs, including activation of the complement cascade and several elements of innate immunity (i.e. granulocytes and monocytes as well as naturally occurring antibodies), play a crucial role in both initiation and execution of the egress of HSPCs from BM into PB [3, 7]. Mice deficient in the mannan-binding lectin (MBL) pathway, which is required for proper activation of the proximal part of the complement cascade [8, 9], as well as mice deficient in the fifth component of the complement cascade (C5), which is a central component of the distal part of the complement cascade by providing C5a and C5a_desArg_ anaphylatoxins [10], are poor mobilizers upon G-CSF administration. The effectiveness of the complement cascade in HSPC mobilization is additionally potentiated by activation of the coagulation cascade [11]. Thus, both the complement and coagulation cascades, which are evolutionarily ancient responses to tissue/organ injuries, are important modulators of stem cell trafficking [11–13]. The crosstalk between these cascades is explained by the fact that thrombin, a product of the coagulation cascade, cleaves C5 to C5a and C5b. It is currently unclear which of the split products then contributes to HSPC mobilization: C5a and its degradation product C5a_desArg_ are known to signal through C5aR (CD88) and potentially C5aR2 (C5L2), whereas C5b and the complement components C6-C9 form the terminal complement complex (TCC). The TCC has also been shown to have biological effects, such as sphingosine-1 phosphate liberation from red blood cells, and is otherwise known as membrane attack complex (MAC) for its characteristic bacterial defense function as a lethal pore [14, 15].

To narrow down on the exact molecular mechanisms we performed studies in C5aR knock-out mice. In a model of HSPC mobilization, we also tested the effect of the plasma-stable, mixed L-RNA/L-DNA aptamer AON-D21, which binds to murine and human C5a/C5a_desArg_ with picomolar affinity and efficiently inhibits their interaction with both C5a receptors [16, 17]. L-Aptamers (Spiegelmers), such as AON-D21, are oligonucleotides identified through repeated rounds of in vitro selection, have been generated to bind to various molecular targets and often exhibit molecular recognition properties that surpass those of traditional antibodies [18, 19]. Importantly, L-aptamers are non-immunogenic and not susceptible to ubiquitous plasma nucleases that cleave natural, D-configured oligonucleotides and have shown to be safe, well tolerated and efficacious in therapeutic applications [20, 21].

Using these two approaches we present novel evidence which confirms the critical role of the C5a/C5a_desArg_–C5aR axis in the pharmacological HSPC mobilization process.

## Materials and Methods

### Animals

In our experiments we employed pathogen-free, 6- to 8-week-old C57BL/6J wild-type mice (WT) and C5ar1^tm1Cge^/J (C5aR-KO) mice of both sexes that were maintained in our animal facility. The Animal Care and Use Committee of the University of Louisville (Louisville, KY, USA) approved the animal studies.

### Mobilization

We performed two different mobilization studies. In the first, experimental mice (18 WT and 18 C5aR-KO in total) were injected subcutaneously (s.c.) with 250 μg/kg G-CSF (Amgen, Thousand Oaks, CA, USA) daily for 3 days (short mobilization) or 6 days (long mobilization) and one dose of 5 mg/kg AMD3100 (Sigma–Aldrich, St Louis, MO, USA) injected intraperitoneally (i.p.). At 6 h after the last G-CSF administration or at 1 h after AMD3100 injection the mice were bled from the retro-orbital plexus for hematology analysis. Briefly, 50 μl of PB were drawn into EDTA-coated Microvette tubes (Sarstedt Inc., Newton, NC, USA) and run within 2 h of collection on a HemaVet 950FS hematology analyzer (Drew Scientific Inc., Oxford, CT, USA). PB was then obtained from the vena cava with a 25-gauge needle and a 1-ml syringe containing 50 μl of 100 mM ethylenediaminetetraacetic acid (EDTA; Quality Biological Inc., Gaithersburg, MD, USA). Mononuclear cells (MNCs) from PB were obtained by hypotonic lysis of RBCs in BD Pharm lysing buffer (BD Biosciences, San Jose, CA, USA) and used for further FACS analysis and clonogenic in vitro assays.

In the second mobilization protocol, WT mice (12 in total) were mobilized with G-CSF (3 days, 100 µg/kg, s.c.) in the absence or presence of the anti-C5a L-aptamer AON-D21. Control mice were injected with vehicle or vehicle combined with reversed AON-D21 (revAON-D21), a control L-aptamer with the same nucleotide composition as AON-D21 that does not bind C5a. At 6 h after the last G-CSF injection, the mice were bled from the orbital plexus for hematology analysis, and PB was obtained from the vena cava as described above.

### Analysis of PB Cells by Flow Cytometry

The following monoclonal antibodies were used to perform staining of Sca-1^+^ c-Kit^+^ Lin^−^ (SKL) cells and Sca-1^+^ CD45^+^ Lin^−^ hematopoietic stem cells (HSCs): FITC–anti-CD117 (also known as c-Kit, clone 2B8; BioLegend, San Diego, CA, USA) and PE–Cy5–anti-mouse Ly-6 A/E (also known as Sca-1, clone D7; eBioscience, San Diego, CA, USA). All anti-mouse lineage marker (Lin) antibodies, including anti-CD45R/B220 (clone RA3-6B2), anti-Ter-119 (clone TER-119), anti-CD11b (clone M1/70), anti-T cell receptor β (clone H57-597), anti-Gr-1 (clone RB6-8C5), anti-TCRγδ (clone GL3), and anti-CD45 (clone 30-F11), were conjugated with PE and purchased from BD Biosciences (San Jose, CA, USA). Staining was performed in RPMI-1640 medium containing 2% FBS. All monoclonal antibodies (mAbs) were added at saturating concentrations, and the cells were incubated for 30 min on ice, washed twice, and analyzed with an LSR II flow cytometer (BD Biosciences, San Jose, CA, USA).

### Evaluation of HSPC Mobilization

For evaluation of circulating colony-forming unit-granulocyte/macrophage (CFU-GM) and SKL cells, the following formulas were used: (number of white blood cells per µl [WBCs/µl]) x number of CFU-GM colonies)/number of WBCs plated = number of CFU-GM per μl of PB; and (number of WBCs per µl x number of SKL cells)/number of gated WBCs = number of SKL cells per μl of PB.

### Clonogenic In Vitro Assay

RBCs from PB were lysed with BD Pharm lysis buffer (BD Biosciences, San Jose, CA, USA). Nucleated cells were subsequently washed twice and counted, and 1 × 10^6^ cells were resuspended in human methylcellulose base medium provided by the manufacturer (R&D Systems, Minneapolis, MN, USA) supplemented with 25 ng/ml recombinant murine granulocyte/ macrophage colony-stimulating factor (mGM-CSF; PeproTech, Rocky Hill, NJ, USA) and 10 ng/ml recombinant murine interleukin 3 (mIL-3; PeproTech). Cultures were incubated for 7 to 14 days (37 °C, 95% humidity, and 5% CO_2_), and the numbers of CFU-GM colonies were scored using an inverted microscope (Olympus, Center Valley, PA, USA). Final results were recalculated based on the number of PBMNCs per 1 μl of PB. Each clonogenic test was performed in duplicate.

### Degranulation Assays

Gr-1^+^ cells were isolated from the BM of adult WT and C5aR-KO mice. Briefly, the BM was flushed from femurs, and the population of total nucleated cells was obtained after lysis of red blood cells (RBCs) using 1 × BD Pharm lysis buffer (BD Biosciences, San Jose, CA, USA). The cells were subsequently stained with PE–anti-Gr-1 antibodies (anti-Ly-6G and -Ly-6C, clone RB6-8C5, BD Biosciences, San Jose, CA, USA) for 30 min in medium containing 2% fetal bovine serum. The cells were then washed, resuspended in RPMI-1640 medium, and sorted using a Moflo XDP cell sorter (Beckman Coulter, Indianapolis, IN, USA). The sorted Gr-1^+^ cells were then resuspended and starved in medium RPMI-1640 plus 0.5% BSA (2 × 10^6^ cells per 400 μl medium) overnight at 37 °C. Next, cells were stimulated by adding G-CSF (100 ng/ml), AMD3100 (3 μM), or medium alone as control and incubated for 6 h at 37 °C. The cells were centrifuged, and conditioned media (CM) were collected and analyzed. Myeloperoxidase (MPO) activity was determined using 3,3′,5,5′-tetra-methylbenzidine (Sigma–Aldrich). Briefly, 20-μl samples were combined with 100 μl of 3,3′,5,5′-tetramethylbenzidine substrate solution, the plate was incubated at 37 °C for 20 min, and the absorption was then measured at 450 nm to estimate myeloperoxidase activity. Elastase activity was measured using the EnzChek Elastase Assay kit, according to the manufacturer’s instructions (Life Technologies, Carlsbad, CA, USA). Briefly, 100 μl of fresh sample was incubated with 100 μl of substrate solution (with 25 μg/ml DQ elastin) for up to 4 h at room temperature in the dark, and the resulting fluorescence was recorded at 515 nm emission following 505 nm excitation. All activity assays were performed in triplicate in 96-well plates and analyzed with a Beckman Coulter DTX 880 multimode detector.

### Statistical Analysis

All results are presented as mean ± SD. Statistical analysis of the data was done using Student’s t-test for unpaired samples (Excel, Microsoft Corp., Redmond, WA, USA) with a value of *p* ≤ 0.05 considered significant.

## Results

### C5aR-Deficient Mice are Poor Mobilizers

It has been previously reported that C5-deficient mice are poor mobilizers [10]. The cleavage of C5 generates both C5a and C5a_desArg_, which are important anaphylatoxins [3], and C5b, which is required for the last step of the distal complement cascade and formation of the C5b-C9 complex (also known as the membrane attack complex) [14, 15]. To assess directly the involvement of the C5a/C5a_desArg_–C5aR axis, we performed mobilization in C5aR-KO mice.

**Fig. 1:**
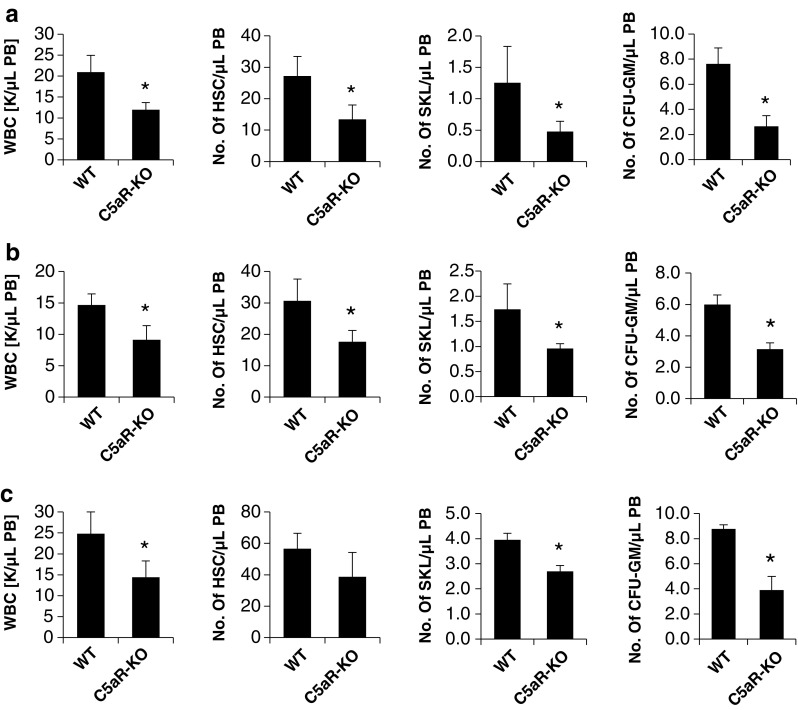
C5aR-KO mice mobilize poorly after AMD3100 or G-CSF administration. Peripheral blood mononuclear cells were isolated from WT and C5aR-KO mice after administration of AMD3100 (5 mg/kg) (**a**) or of 3 days (**b**) or 6 days (**c**) of G-CSF (250 μg/kg per day, subcutaneously). The numbers of WBCs, SKL (Sca-1^+^ c-Kit^+^ Lin^−^) cells, HSCs (Sca-1^+^ CD45^+^ Lin^−^), and CFU-GM clonogenic progenitors were evaluated in PB samples. Results from two separate experiments are pooled together. *P ≤ 0.05

As shown in Fig. 1, we found that C5aR-KO mice mobilized poorly in response to AMD3100 administration (Fig. 1a) as well as to a short (3-day) (Fig. 1b) or a long (6-day) (Fig. 1c) G-CSF administration. This effect was observed throughout all cell populations that we analysed. Specifically, the numbers of WBCs, SKL (Sca-1^+^ c-Kit^+^ Lin^−^) cells, HSCs (Sca-1^+^ CD45^+^ Lin^−^), and CFU-GM clonogenic progenitors were mobilized less efficiently into PB in C5aR-KO animals.

### Gr-1^+^ Cells from C5aR-Deficient Mice Show a Defect in Degranulation

From observations in C5-deficient mice we had previously deduced that C5a and C5a_desArg_ strongly chemoattract granulocytes and monocytes during the mobilization process and that as these first cells egress from BM they pave the way for egress of HSPCs [10, 22]. We also postulated that C5a/C5a_desArg_-mediated activation of the release of proteolytic and lipolytic enzymes from Gr-1^+^ leucocytes impairs the SDF-1–CXCR4 and VCAM-1–VLA4 axes, which are involved in retention of HSPCs in BM niches [23].

**Fig. 2:**
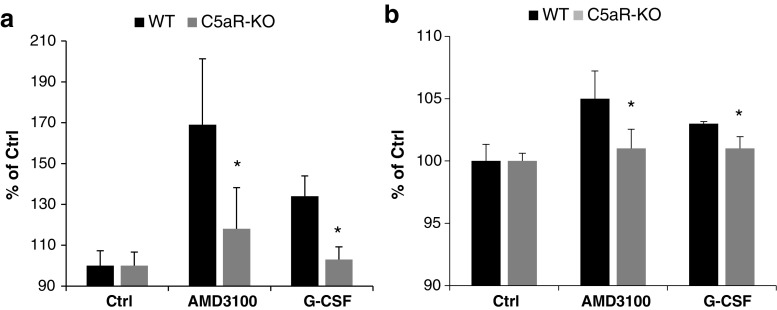
Gr-1^+^ cells isolated from C5aR-KO mice exhibit defective pro-mobilizing properties. Degranulation of BM-derived Gr-1^+^ cells sorted from WT and C5aR-KO mice was measured by MPO release from the cells during the degranulation process (**a**) and by elastase activity (**b**). The results are combined from two independent experiments and show changes as a percentage of control. *P ≤ 0.05

Figure 2 shows that degranulation of Gr-1^+^ cells isolated from C5aR-KO mice is impaired, as evaluated by measuring the release of myeloperoxidase (Fig. 2a) and elastase (Fig. 2b) in response to stimulation by AMD3100 or G-CSF. Poor responsiveness of Gr-1^+^ cells from C5aR-KO mice to stimulation by the CXCR4 antagonist AMD3100 and G-CSF, which activates the G-CSF receptor, suggests that both compounds need functional C5aR for optimal degranulation and release of proteolytic enzymes. This observation, however, requires further verification. Of note, we have shown in the past that Gr-1^+^ cells become directly activated in response to AMD3100 exposure [6].

### In Vivo Inhibition of C5a by Spiegelmer AON-D21 Impairs Mobilization of HSPCs

**Fig. 3:**
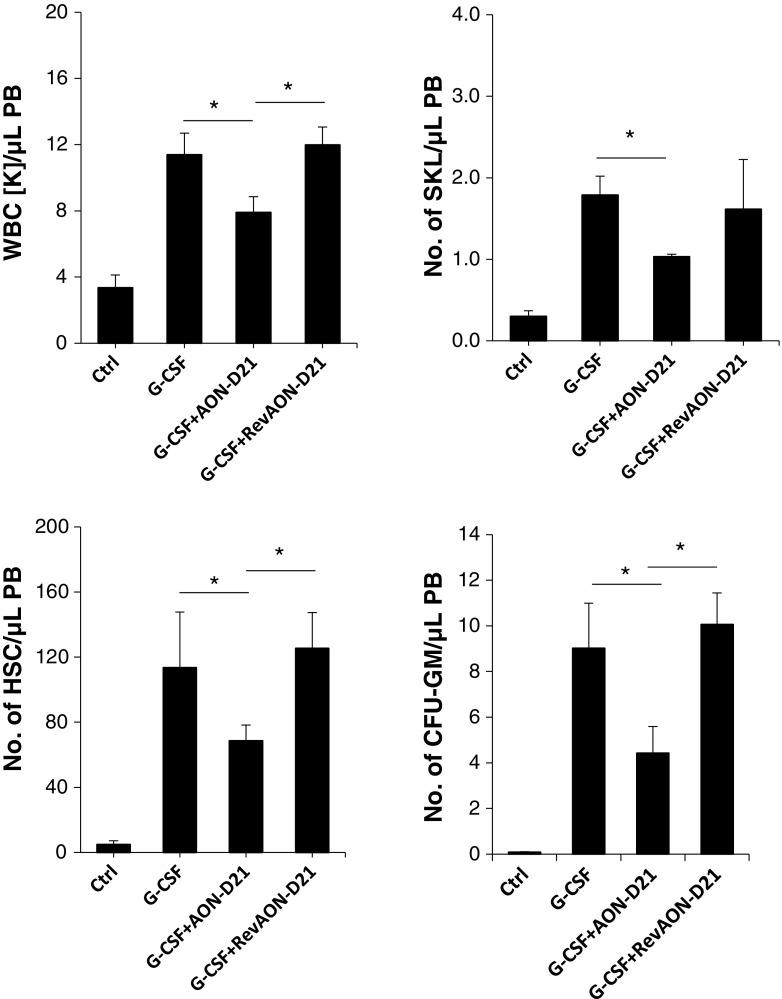
Reduced mobilization in WT mice exposed to the AON-D21 Spiegelmer, which neutralizes C5a. Mononuclear cells were isolated from WT mice after 3 days of administration of G-CSF (100 μg/kg per day, s.c.) in the absence or presence of AON-D21. Control mice were injected with vehicle and vehicle combined with reverse AON-D21 (revAON-D21). The numbers of WBCs, SKL (Sca-1^+^ c-Kit^+^ Lin^−^) cells, HSCs (Sca-1^+^ CD45^+^ Lin^−^), and CFU-GM clonogenic progenitors were evaluated in PB samples. Results from two separate experiments are pooled together. *P ≤ 0.05

We previously reported that C5-deficient mice, which do not generate the C5 cleavage fragments C5a and C5a_desArg_, are poor mobilizers. To better address the role of C5a, we employed the C5a-binding L-aptamer AON-D21to neutralize murine C5a in mobilization experiments (Fig. 3).

As shown in Fig. 3, we found that WT mice exposed to AON-D21, but not to the control L-aptamer revAON-D21 (with the same nucleotide composition as AON-D21 but not binding to C5a), mobilized poorly in response to G-CSF administration. Specifically, the numbers of WBCs, SKL (Sca-1^+^ c-Kit^+^ Lin^−^) cells, HSCs (Sca-1^+^ CD45^+^ Lin^−^), and CFU-GM clonogenic progenitors were mobilized less efficiently in mice if C5a was inhibited by AON-D21.

## Discussion

The seminal contribution of this work is direct evidence that the C5a receptor is required for the optimal mobilization of HSPCs. We demonstrate that C5aR-KO mice are poor mobilizers in response to administration of the CXCR4 antagonist AMD3100 as well as both short (3-day) and long (6-day) administration of G-CSF. Moreover, these observations were subsequently confirmed in a model of HSPC mobilization employing the C5a-binding and neutralizing L-aptamer AON-D21.

Understanding the mechanisms that govern mobilization of HSPCs is crucial for optimizing protocols for hematopoietic transplantation in which cells isolated from mobilized PB are employed [24]. Unfortunately, in autologous transplant settings ~ 10% of normal patients and ~ 25% of patients after chemotherapy are poor mobilizers that do not respond efficiently to currently recommended mobilization protocols [25, 26]. Therefore, it is important to develop more efficient mobilization protocols in order to harvest the required number of HSPCs for hematopoietic transplantation.

The mobilization of HSPCs is an important and evolutionarily ancient biological process that is regulated by several redundant mechanisms. Specifically, the mobilization process involves the chemokine SDF-1, leukocyte adhesion molecules and components of innate immunity, including Gr-1^+^ granulocytes and monocytes along with activation of the complement cascade [3, 10, 12].

In support of a role for innate immunity in HSPC mobilization, we previously reported that the complement cascade becomes activated in bone marrow (BM) during AMD3100 or granulocyte colony-stimulating factor (G-CSF) mobilization of HSPCs [6, 12]. The activation of Gr-1^+^ granulocytes and monocytes by mobilizing agents and C5 cleavage fragments leads to the release of both proteolytic enzymes [11] and lipolytic enzymes [23] that disrupt the SDF-1–CXCR4 and VCAM-1–VLA-4 retention axes that anchor HSPCs in their BM niches [1]. In this context, it was important to more thoroughly explore the role of the complement cascade in modulating the function of Gr-1^+^ granulocytes and monocytes in BM, as they are the first cells that egress from BM into PB and pave the way for HSPCs to cross the BM–PB endothelial barrier [10, 27]. This event occurs in response to an increase in C5a level in PB due to C5 cleavage and activation. It is well known that C5a is a potent activator and chemoattractant for Gr-1^+^ cells [3, 10], and we postulated that activation/cleavage of C5 releases the C5a anaphylatoxin, which directly stimulates granulocytes to release proteolytic and lipolytic enzymes as well as chemoattracts these cells and promotes their egress into PB [6, 10].

While enhancing mobilization is of clinical importance for poor mobilizers, inhibition of increased motility of HSPCs could be of therapeutic importance in patients suffering from paroxysmal nocturnal hemoglobinuria (PNH) [28]. It is well known that in PNH patients, pathological activation of the complement cascade leads to accelerated lysis of erythrocytes due to formation of C5b-C9 (also known as the membrane attack complex, MAC). As a consequence of this unwanted activation, the bioactive lipid sphingosine-1-phosphate (S1P) is released from lysed erythrocytes, and this major chemoattractant of HSPCs [15] preferentially increases motility of PNH-affected HSPCs in the bone marrow (BM) microenvironment [28]. This increased motility promotes their expansion by enabling them to outcompete normal HSPCs for BM niches. To inhibit the complement cascade activation that initiates this sequence of events, a monoclonal antibody, eculizumab, is currently employed to block C5 cleavage via the C5 convertase. Since eculizumab, as recently reported, may not be able to efficiently block C5 cleavage by serine proteases such as thrombin [29] and close bidirectional interactions between the coagulation cascade and the complement system are well described [11], AON-D21 could play a supportive role. Moreover, inhibitors of the C5a-C5aR axis could also find application in other situations when complement cascade is hyperactivated such as for example in Atypical Hemolytic Uremic Syndrome (aHUS) [30].

It is known that, in addition to C5aR, C5 cleavage fragments activate the second C5a receptor, C5aR2 (also known as C5L2 or G protein-coupled receptor 77) [31]. While we demonstrate here an important role for C5aR, we cannot exclude additional involvement of C5aR2 in mobilization, which may somewhat compensate for the lack of C5aR. In fact, mobilization, even if severely impaired, still occurs in C5aR-KO mice that carry the normal C5aR2. This finding requires further study in mice deficient in C5aR2 and in both receptors (C5aR-KO plus C5aR2-KO). It would also be interesting to directly compare C5 knock out with C5aR knock out as to elucidate the contribution of the terminal complement complex. Of note; the anti-C5a L-aptamer AON-D21, employed in this study, prevents binding of C5a to both receptors and is equally potent as C5aR knock-out.

Another important observation is the *in vivo* effectiveness of the anti-C5a L-aptamer AON-D21 targeting the distal part of the complement cascade, which, as demonstrated in our current work, significantly diminished the mobilization of HSPCs. This further clarifies our previous results on the role of C5 cleavage fragments in the egress of HSPCs from BM into PB [10, 12, 22].

In summary, by employing complementary strategies we confirmed the critical role of the C5a/C5a_desArg_–C5aR axis in the mobilization of HSPCs. Further studies, however, are important to address the contribution of a second C5a and C5a_desArg_ receptor, namely the C5a2 receptor that is intact in C5aR-KO mice, as well as involvement of the C5b-C9 complex (membrane attack complex; MAC). This latter complex could increase the PB level of a potent chemoattractant for HSPCs, sphingosine-1-phosphate, which is released from red blood cells [15]. In fact in our previous paper we demonstrated that inhibition of mobilization process in C5 deficient mice was more profound (~ 85%) [10] as compared herein to C5aR^−/−^ animals (~ 45%). This suggests a significant contribution of MAC to mobilization of HSPCs and future studies with C6^−/−^ mice will allow to address this issue. We propose also that novel drugs that inhibit the C5a–C5aR axis, such as the L-aptamer AON-D21 or C5aR antagonists, can find practical application in the treatment of PNH, aHUS or other clinical states when complement cascade is activated [32] in addition to eculizumab [29, 30, 33, 34] or compstatin [35, 36].
